# Development of 16 Microsatellite Markers for Prince’s Pine, *Chimaphila japonica* (Pyroleae, Monotropoideae, Ericaceae)

**DOI:** 10.3390/ijms13044003

**Published:** 2012-03-23

**Authors:** Zhenwen Liu, Qianru Zhao, Jing Zhou, Hua Peng, Junbo Yang

**Affiliations:** 1 Key Laboratory of Biodiversity and Biogeography, Kunming Institute of Botany, Chinese Academy of Sciences, Kunming 650201, China; E-Mails: liuzw@mail.kib.ac.cn (Z.L.); zhaoqianruzhao@126.com (Q.Z.); hpeng@mail.kib.ac.cn (H.P.); 2 Graduate School of Chinese Academy of Sciences, Beijing 100049, China; 3 School of Pharmaceutical Science & Yunnan Key Laboratory of Pharmacology for Natural Products, Kunming Medical University, 1168 Western Chunrong Road, Yuhua Street, Chenggong New City, Kunming 650500, Yunnan, China; E-Mail: zhoujing520@gmail.com; 4 Germplasm Bank of Wild Species in Southwest China, Kunming Institute of Botany, Chinese Academy of Science, Kunming 650201, Yunnan, China

**Keywords:** *Chimaphila japonica*, microsatellite marker, polymorphism, population genetics

## Abstract

The perennial evergreen herb, *Chimaphila japonica* is found exclusively in East Asian temperate coniferous or sometimes in deciduous forests. By using the Fast Isolation by Amplified Fragment Length Polymorphism (AFLP) of Sequences Containing repeats (FIASCO) protocol, 20 microsatellite primer sets were identified in two wild populations. Of these primers, 16 displayed polymorphisms and 4 were monomorphic. The number of alleles per locus ranged from one to six among populations, values for expected and observed heterozygosities ranged from 0.000 to 0.848 and from 0.000 to 1.000, respectively. The new SSR markers will be useful in obtaining estimates of population-level genetic diversity and in phylogeographic studies of *C. japonica*.

## 1. Introduction

Members of the small genus *Chimaphila* are evergreen herbs, found in patches on the floor of coniferous or sometimes deciduous forests across the Northern Hemisphere. *Chimaphila japonica*, the focal species of the present study, is exclusively distributed in East Asia [1]. Floristic studies suggested that the forest area in the East Asiatic Kingdom could be divided into two subkingdoms, namely the Sino-Japanese Forest subkingdom and the Sino-Himalayan Forest subkingdom [2,3]. It has long been thought that the formation of these spatiotemporal patterns of biodiversity correlates with physical environmental changes resulting from the Himalaya-Tibet Plateau uplifting [4,5]. However, the nature and extent of this correlation remains unexplained. Intensive biological observation of widespread species at the population level may provide insights into the biogeography of this region. As part of our broader research on the systematic and evolutionary history of *Chimaphila*, in this study we isolate and characterize fast-evolving codominant microsatellite markers to gain a better understanding of the relationships among populations of *C. japonica*, and the amount and geographic distribution of its genetic diversity.

## 2. Results and Discussion

A total of 260 positive clones were captured: among these 158 clones (61%) were found to contain simple sequence repeats (SSR). Of the 125 primer sets, 20 microsatellite loci successfully amplified in *C. japonica*; 16 of them were found to be polymorphic and the remaining four were monomorphic (Table 1).

In the Kangding population, the number of alleles per locus ranged from one to six, with an average of 2.9, and the expected and observed heterozygosities ranged from 0.000 to 0.848 and from 0.000 to 1.000, respectively. In the Kunming population, the number of alleles per locus varied between one and six, with an average of 2.6, and the expected and observed heterozygosities ranged from 0.000 to 0.775 and from 0.000 to 1.000, respectively (Table 2).

An excess of homozygotes was simultaneously observed for seven loci (Chim009, Chim011, Chim029, Chim037, Chim069, Chim110 and Chim122) in both populations (Table 2), which were possibly caused by the limited sample size. And the possibility of the occurrence of some null alleles at these loci could not be completely ruled out. Compared with the one monomorphic locus (Chim110) found in the Kangding population, four loci (Chim035, Chim037, Chim054 and Chim107) were observed in the Kunming population (Table 2). This difference is possibly caused by the areas’ different population histories. The Qinghai-Tibetan Plateau distributed Kangding population may have been extensively influenced by the uplift of the Himalayas and the Tibetan Plateau. However, the Yungui Plateau-distributed Kunming population may have experienced simpler geological events, and notably, it is the most southern distribution as far as we know, having a relatively short evolutionary history.

## 3. Experimental Section

Genomic DNA samples of *C. japonica* were extracted from silica-gel-dried leaves of two different individuals using a modified hexadecyltrimethylammonium bromide (CTAB) method [6]. The extracted DNA was dissolved in ddH_2_O. The fast isolation by AFLP of Sequences Containing repeats (FIASCO) was performed in this study [7]. Total genomic DNA (approximate 250 ng) was completely digested with 2.5 U of *Mse*I restriction enzyme (New England Biolabs, Beverly, MA, USA), and then ligated to an *Mse*I AFLP adaptor (5′-TAC TCA GGA CTC AT-3′/5′-GAC GAT GAG TCC TGA G-3′) using T4 DNA ligase (Fermentas, Burlington, ON, Canada). The digested-ligated fragments were diluted in a ratio of 1:10, and 5 μL were used amplification reaction with *Mse*I-*N* primer (5′-GAT GAG TCC TGA GTA AN-3′). The amplified DNA fragments (200–800 bp) were enriched by magnetic bead selection with 5-biotinylated (AG)_15_ and (AC)_15_ probes, respectively. The recovered DNA fragments were reamplified with *Mse*I-*N* primers. The PCR products were purified using the EZNA Gel Extraction Kit (Omega Bio-Tek, Guangzhou, China), and were ligated into a pGM-T vector (Tiangen, Beijing, China), and then transformed into *E. coli* strain DH5α competent cells (TaKaRa, Dalian, Liaoning, China). The positive clones were picked out and tested using vector primers T7/SP6 and primer (AC)_10_/(AG)_10_ respectively to select appropriate fragments which contained SSRs. In other words, a set of tested PCRs included three reactions was performed using T7 and SP6, T7 and (AC)_10_, (AC)_10_ and SP6 as primers, respectively. The second set of tested PCRs was tested using T7 and SP6, T7 and (AG)_10_, (AG)_10_ and SP6 as primers, respectively. All of these PCR reactions had the same conditions: 94 °C for 4 min followed by 35 cycles at 94 °C for 45 s, 52 °C for 45 s, 72 °C for 1 min, and a final extension step at 72 °C for 3 min. The selected positive clones were sequenced using an ABI PRISM 3730XL DNA sequencer (Applied Biosystems, Foster City, CA, USA). Sequences containing simple sequence repeats and enough flanking regions were selected for primer design using Primer Premier 5.0 [8].

The designed Primer pairs were assessed in 24 individuals of *C. japonica* pooled from two natural populations (KD, Kangding, Sichuan: 30°09′N, 102°09′E, 2857 m a.s.l. and KM, Kunming, Yunnan: 25°11′N, 102°44′E, 2345 m a.s.l.). Herbarium vouchers were deposited in the Kunming Institute of Botany, Chinese Academy of Science (code LZW 0128-0151). The PCR reactions were performed in 20 μL of reaction volume containing 30–50 ng genomic DNA, 0.35 μM of each primer, 10 μL 2× Taq PCR MasterMix (Tiangen; 0.1 U Taq Polymerase/μL, 0.5 mM dNTP each, 20 mM Tris-HCl (pH 8.3), 100 mM KCl and 3 mM MgCl_2_). PCR amplifications were conducted under the following conditions: 94 °C for 3 min followed by 32 cycles at 94 °C for 30 s, the annealing temperature for each specific primer (optimized for each locus, Table 1) for 30 s and 72 °C for 45 s with a final extension step at 72 °C for 3 min. The amplification products were separated and visualized using a QIAxcel capillary gel electrophoresis system (QIAGEN, Irvine, CA, USA).

The number of alleles per locus, observed heterozygosity (*H*_O_), and expected heterozygosity (*H*_E_) were estimated in POPGENE version 1.31 [9].

## 4. Conclusions

All 16 microsatellite loci described here showed a useful degree of polymorphism at the population level and will be useful to reveal the history of *C. japonica* in East Asia, population dynamics, and genetic structure.

## Figures and Tables

**Table 1:** Characteristics of 20 microsatellite loci developed in *Chimaphila japonica.*

Locus	Primer sequence (5′–3′)	Repeat motif	Size range (bp)	*T*_a_ (°C)	GenBank Accession No.
Chim004 *	F: CGTCACTTCTAACTCAAATCC R: TCATTGGGTGTTGTTGTGT	(AC)_31_	249–297	50	JQ392547
Chim009 *	F: CGCTTCTCTTCACAGCTATT R: ACATCCCTTAGTTTACCTCAG	(AC)_24_	240–272	52	JQ392548
Chim011 *	F: CCATCCATTTCCTCAATCAT R: TTAGGCTTACCACCAAGAAC	(AG)_6_CT (AG)_6_	157–191	50	JQ392549
Chim012 *	F: TTTACTTTCTCTTCTTCCCC R: TTTTTGCTTGCTTTACATCT	(AG)_19_	176–192	40	JQ392550
Chim025 *	F: TATTTCACATAGACGAGGGT R: GACGAACACAGTAACGGAC	(AG)_18_	133–151	52	JQ392551
Chim029 *	F: GTCAGGAAACAAGCGAGGAA R: CATCAGTTGAGAAATGGGTA	(AC)_23_	291–357	52	JQ392553
Chim035 *	F: TCCAGGAGTGTGAGACATAG R: TACCGAAGGCACGATAAACA	(AC)_24_	148–160	50	JQ392554
Chim037 *	F: TTCTCAAAAGCCAATCACAC R: CCTCATTCCATCCGAAGTCC	(AC)_9_	125–141	46	JQ392555
Chim054 *	F: TGAGGAGACGAACACAACAA R: CATCCATCCAAAGGCTCTAC	(AC)_8_	195–213	52	JQ392556
Chim064 *	F: CGCACGCATTTGTATTTA R: GGTGTATGGAACATCACGCA	(AG)_10_ (TG)_12_	127–155	48	JQ392557
Chim069 *	F: TGCTCTAACAGATATGCGTG R: CGTGTATGTAGAATGGGAG	(AC)_10_	76–86	48	JQ392558
Chim092 *	F: ATGAGAAGAAGTCTGGGAGAG R: CATAGTGAACCACAAAGCC	(TG)_9_(AG)_10_	87–101	50	JQ392560
Chim107 *	F: ACCATCTCAAACTTCACTAAG R: CCATTCTCTCCCTCATTACC	(AC)_8_	237–249	52	JQ432377
Chim110 *	F: GCTTTATTTTGTCTTAGGGT R: AAACTTGAACTTGAATGGAA	(AG)_15_	197–205	48	JQ432378
Chim119 *	F: CAGGATGCTCTTTGAATTAG R: AAGGTATGAGAAGCGAAGGG	(AG)_23_ (AC)_11_	143–171	50	JQ432379
Chim122 *	F: GACCAAGGGTGGACCAAGAG R: AAACAACTTACGCATAGAAA	(AG)_15_	107–125	48	JQ432380
Chim27	F: TTTCTCATCTCTCCCTACTG R: GAGTTTATTCCTTATGCCTT	(AG)_4_TT(AG)-(AC)_5_	100	48	JQ392552
Chim80	F: GGCATACAACATCTAACGAG R: TCTCTCAAGGTTTTGCTTC	(AG)_17_	254	50	JQ392559
Chim97	F: TGAGATGCCCATCCTCTCTG R: CGAACAAATCACACAAATTCC	(AC)_8_	262	52	JQ392561
Chim99	F: ATCCCAGATATACGGCACAC R: GAACACCAAAAGGCTATGTA	(AG)_12_	221	52	JQ392562

* Displayed polymorphisms in *Chimaphila japonica*;

*T*a, PCR annealing temperature.

**Table 2:** Locus-specific measures of genetic diversity of two populations of *Chimaphila japonica*.

	Population KD (*N* = 12)			Population KM (*N* = 12)		

Locus	*N*_A_	*H*_E_	*H*_O_	*N*_A_	*H*_E_	*H*_O_
Chim004	5	0.688	1.000	4	0.775	1.000
Chim009	3	0.565	0.000	4	0.670	0.083
Chim011	3	0.649	0.083	2	0.083	0.083
Chim012	4	0.576	0.333	6	0.775	0.250
Chim025	4	0.703	0.333	4	0.544	0.333
Chim029	3	0.594	0.000	3	0.652	0.000
Chim035	2	0.228	0.250	1	0.000	0.000
Chim037	2	0.228	0.083	1	0.000	0.000
Chim054	3	0.638	1.000	1	0.000	0.000
Chim064	3	0.565	0.167	3	0.489	0.583
Chim069	2	0.159	0.000	2	0.507	0.000
Chim092	2	0.083	0.083	2	0.464	0.667
Chim107	2	0.228	0.250	1	0.000	0.000
Chim110	1	0.000	0.000	2	0.159	0.167
Chim119	6	0.848	0.250	4	0.736	0.417
Chim122	2	0.083	0.083	2	0.083	0.083

*N* = population sample size; *N*_A_ = number of alleles revealed; *H*_E_: expected heterozygosity; *H*_O_: observed heterozygosity; Population KD (Kangding, Sichuan: 30°09′N, 102°09′E, 2857 m a.s.l.); Population KM (Kunming, Yunnan: 25°11′N, 102°44′E, 2345 m a.s.l.).
